# Transcriptome analysis reveals the roles of phytohormone signaling in tea plant (*Camellia sinensis* L.) flower development

**DOI:** 10.1186/s12870-022-03853-w

**Published:** 2022-10-04

**Authors:** Xiaohan Xu, Jing Tao, Anqi Xing, Zichen Wu, Yuqin Xu, Yi Sun, Jiangyuan Zhu, Xiang Dai, Yuhua Wang

**Affiliations:** 1 College of Horticulture, Nanjing Agricultural University, Nanjing, 210095 China; 2 Tea Research Institute of Tianmu Lake in Liyang Changzhou, Changzhou, 213300 China

**Keywords:** *Camellia sinensis*, Flower development, Transcriptome (RNA-seq), Phytohormone

## Abstract

**Background:**

Tea plant (*Camellia sinensis* (L.) O. Kuntze) is an important economic tea crop, but flowering will consume a lot of nutrients of *C. sinensis*, which will seriously affect the nutritional growth of *C. sinensis*. However, there are few studies on the development mechanism of *C. sinensis* flower, and most studies focus on a single *C. sinensis* cultivar.

**Results:**

Here, we identified a 92-genes’ *C. sinensis* flower development core transcriptome from the transcriptome of three *C. sinensis* cultivars ('BaiYe1', 'HuangJinYa' and 'SuChaZao') in three developmental stages (bud stage, white bud stage and blooming stage). In addition, we also reveal the changes in endogenous hormone contents and the expression of genes related to synthesis and signal transduction during the development of *C. sinensis* flower. The results showed that most genes of the core transcriptome were involved in circadian rhythm and autonomous pathways. Moreover, there were only a few flowering time integrators, only 1 *HD3A*, 1 *SOC1* and 1 *LFY*, and *SOC1* played a dominant role in the development of *C. sinensis* flower. Furthermore, we screened out 217 differentially expressed genes related to plant hormone synthesis and 199 differentially expressed genes related to plant hormone signal transduction in *C. sinensis* flower development stage.

**Conclusions:**

By constructing a complex hormone regulation network of *C. sinensis* flowering, we speculate that *MYC*, *FT*, *SOC1* and *LFY* play key roles in the process of endogenous hormones regulating *C. sinensis* flowering development. The results of this study can a provide reference for the further study of *C. sinensis* flowering mechanism.

**Supplementary Information:**

The online version contains supplementary material available at 10.1186/s12870-022-03853-w.

## Background

The tea plant (*Camellia sinensis* (L.) O. Kuntze) is a cash crop with significant importance, which is grown commercially in more than 60 countries, including China [1]. Tea, the most popular non-alcoholic beverage in the world, is frequently made from its leaves and buds. However, the productivity and quality of tea are significantly impacted by the excessive nutrient consumption caused by vigorous reproductive growth [2]. As a result, it is crucial to explore the potential molecular mechanism of flower development and its influencing factors for controlling the flowering of *C. sinensis*, promoting the vegetative growth of *C. sinensis*, and increasing the yield of tea. In the growth and development of plants, flowering is a crucial and intricate process involving a series of physiological and biochemical changes controlled by a large number of genes [3]. Over the past few years, with the rapid advancement of molecular biotechnology, the flowering process has been thoroughly explored in model plants like *Arabidopsis thaliana* and to a lesser extent in a few other plant species [4–7]. In general, the flowering process can be subdivided into three major phases: floral induction, floral meristem formation, and floral organ development [8].

The first phase of plant reproductive growth, known as floral induction, is thought to be the consequence of plants responding to endogenous signals and external environmental stimuli [3]. Currently, research on the model plant *A. thaliana* suggests that at least seven pathways, including photoperiod pathway, gibberellin pathway, autonomous pathway, vernalization pathway, temperature sensitive pathway, aging pathway and trehalose-6-phosphate pathway, may be involved in controlling flower formation in plants [9, 10]. These approaches are both independent and interwoven, and finally through (*FLOWERING LOCUS T*) *FT*, *FLOWERING LOCUS C* (*FLC*), *LEAFY* (*LFY*) and *SUPPRESSOR OF OVEREXPRESSIONOF CONSTANS 1* (*SOC1*) integration genes in flowering to realize the regulation of plant flowering transformation, and formed a complex flowering network with precise regulation function [11, 12].

After floral induction, flowering pathway integration genes can encourage shoot apical meristem (SAM) differentiation into inflorescence meristem (IM) by activating the expression of inflorescence meristem-specific genes. The inflorescence meristem will then differentiate into floral meristem (FM) under the control of flower meristem-specific genes. This process is called flower initiation. *TERMINAL FLOWER1* (*TFL1*) and *EMBRYONIC FLOWER* (*EMF*) are two IM characteristic genes isolated from *A. thaliana*, both of which are flowering inhibition genes [13, 14]. *LFY* and *APETALA1* (*AP1*) are two main FM characteristic genes, which are partially redundant in function and have a superposition effect. They serve the purpose of encouraging the primordia on the side of IM to grow into blooms rather than leaf buds [15].

The activation of floral meristem-specific genes can activate flower organ-specific genes during the blooming metamorphosis of plants, resulting in the creation of floral organs. With the discovery and cloning of more and more genes related to the development of plant floral organs, the development models of plant floral organs have been continuously supplemented and enhanced. From the initial “ABC model” [16], it has developed into “ABCD model” [17], “ABCDE model” [18] and later perfect “tetramer model” [19, 20] (Supplementary Fig. S1). The establishment of these models has laid a foundation for people to study the internal relations between different flower forming genes and the systematic division of flower organ development genes of different plants.

Recently, with the genome sequencing of big-leaf *C. sinensis var. assamica* (CSA) and *C. sinensis* var. *sinensis* (CSS) successively completed, high-quality *C. sinensis* genome information has been obtained [21], which has effectively promoted the in-depth analysis of the structural annotation and function of *C. sinensis* transcriptome, and provided an opportunity to screen the genes of *C. sinensis* flower development and analyze the mechanism of induction, transition and differentiation of *C. sinensis* flower development in the whole genome. Currently, researches on *C. sinensis* flowering had made some progresses, but so far we know little about the complex network regulating flower development. At present, 401 CSS and 356 CSA flowering-related genes have been identified at genome level [3]. Study had shown that photoperiod, gibberellin and sugar pathway were also involved in abiotic stress response, and the photoperiod genes *PRR7* and *GI* and the gibberellin receptor genes *GID1B* and *GID1C* might play an important role in flower induction, as well as *PNF*, *PNY* and *LFY* might play an important role in flower transformation. In this study, the flowers of the three development stages (“young bud stage”, “white bud stage” and “full bloom stage”) of three *C. sinensis* cultivars (‘BaiYe1’, ‘HuangJinYa’ and ‘SuChaZao’) were used as the research objects. The transcriptome analysis of *C. sinensis* flowers had preliminarily constructed a possible flowering regulation network in *C. sinensis*, which provided a reference for further exploring the flowering mechanism of *C. sinensis*, and also provided a theoretical basis for controlling *C. sinensis* flowering, promoting vegetative growth, and increasing tea yield. In addition, we also revealed for the first time the changes in the content of various plant endogenous hormones and the expression of related genes during the flower development of *C. sinensis*, which will help to understand the role and mechanism of endogenous hormones in the flowering of *C. sinensis*.

## Results

### Transcriptomic profiling of flower development in three C. sinensis cultivars

For RNA sequencing (RNA-seq) study, 27 RNA samples from three developmental phases of three cultivars were collected in triplicate. After removing adaptors, low quality and ambiguous reads, the clean rates were all higher than 97.55%, and the Q30 values (sequencing error rate, 1‰) were all over 92.81% (Supplementary Table S4). All clean reads were subsequently mapped onto the reference genome of *C. sinensis* [22]. The total mapped ratio of each sample ranged from 90.21% to 91.57%. (Supplementary Table S4).

By counting the number of expressed genes in each cultivar at each developmental stage, the full data set was first evaluated (Supplementary Fig. S3). In this study, the number of genes with low and medium expression levels (10 > FPKM > 1 and 100 > FPKM > 10, respectively) were significantly decreased after S1 in all cultivars, whereas the number of genes with high and extremely low expression levels (FPKM > 100 and 1 > FPKM > 0, respectively) increased significantly. Notably, the number of genes with FPKM less than 1 (in RNA-seq analyses, genes with a FPKM value no great than 1 are typically considered as not expressed) increased the most, from 13.5% (3,080 ± 315 transcripts) in S1 to 30.4% (6,879 ± 1,027 transcripts) in S2 and 32.3% (7,299 ± 1,357 transcripts) in S3, indicating that the expression of most genes were down-regulated with the development of *C. sinensis* flowers.

**Fig. 1:**
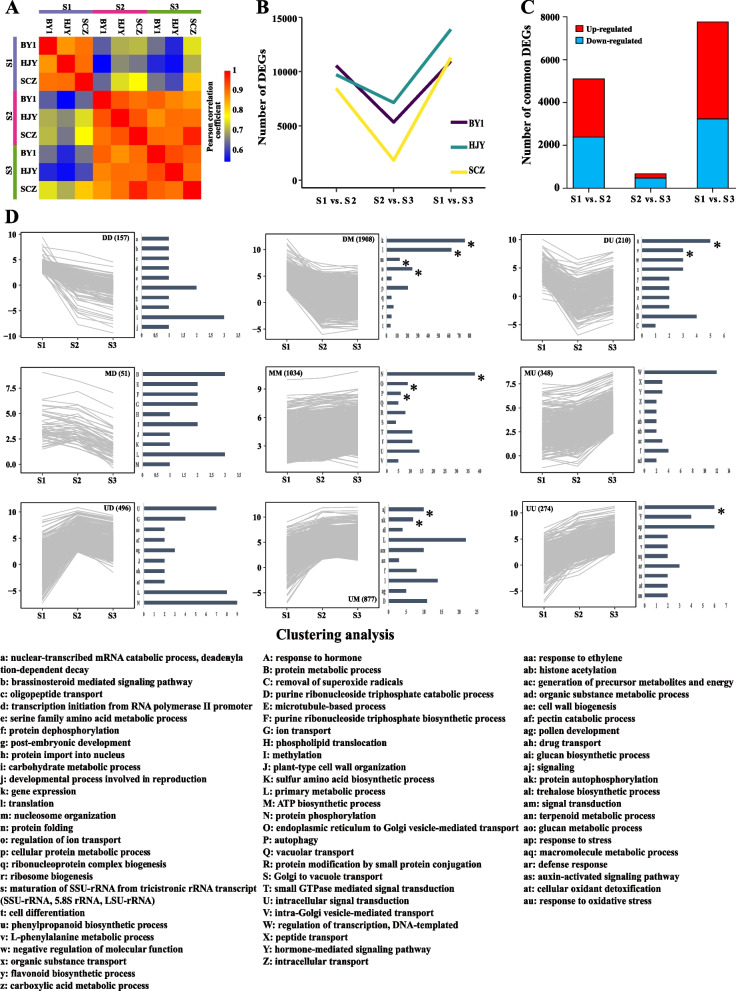
Heatmap of Pearson correlation coefficient matrix during flower development of three *C. sinensis* cultivars. **A** The core transcriptome of *C. sinensis* flower development. The number of DEGs in different development stages of each variety. **B** The number of DEGs commonly up-regulated and down-regulated in three varieties between each pair of development stages. **C** Cluster analysis and GO functional classification of the core flower development transcriptome. **D** BY1 represents the *C. sinensis cv.* ‘BaiYe1’, HJY represents the *C. sinensis cv.* ‘HuangJinYa’, SCZ represents the *C. sinensis cv.* ‘SuChaZao’, S1-S3 represent the young bud stage, white bud stage and full bloom stage of *C. sinensis* flower developmental stages. DEGs represents the differently expression genes. Significantly enriched GO categories (adjusted *P* ≤ 0.01)are represented by asterisks

In order to compare transcriptomes representing the three development stages in each variety, the Pearson correlation coefficient was performed (Fig. 1A). The results showed that apart from the same period, there was also a strong correlation between S2 and S3, regardless of the variety.

### Definition of the core flower development transcriptome

To define genes with similar expression profiles in 3 cultivars during flower development, the differentially expressed genes (DEGs) were firstly identified between each pair of developmental stages (i.e. S1-S2, S2-S3, and S1-S3) using Cuffdiff version 2.0.2 [23]. The number of DEGs of BY1, HJY and SCZ were 14,235, 15,421 and 12,062, respectively, indicating that the development of *C. sinensis* flowers involves transcriptional regulation of a large number of genes (Fig. 1B). The observation of the number of DEGs in each pairwise comparison showed that the dynamics of gene expression regulation were similar among all cultivars. Furthermore, the expression of common DEGs (C-DEGs) in each pair of development stages of the three cultivars was further counted (Fig. 1C), and a total of 8611 C-DEGs were found to be up-regulated or down-regulated in the alignment of at least one group of development stages of the three cultivars. The results showed that in S1 vs. S2, 2338 DEGs were up-regulated and 2717 DEGs were down-regulated. In S2 vs. S3, 477 DEGs were up-regulated and 192 DEGs were down-regulated. In S1 vs. S3, 3240 DEGs were up-regulated and 4523 DEGs were down-regulated.

To establish the core transcriptome of *C. sinensis* flower development, the expression trends of DEGs in adjacent developmental stages were compared to cluster 8611 DEGs that jointly regulate the development of *C. sinensis* flower. After eliminating DEGs with inconsistent expression trends in different cultivars, 5355 DEGs with similar expression trends were identified in all three cultivars, which were classified into 9 clusters (Fig. 1D).

Cluster 1 contained 157 DEGs, whose expression were continuously down-regulated during the whole development of *C. sinensis* flower. The functional annotation mainly includes “DNA-directed RNA polymerase II subunit 4”, “Serine hydroxymethyl transferase 4”, “Mitochondrial import inner membrane transferase” and “homeobox protein ATH1”.

Cluster 2 contained 1908 DEGs, and their expression were down-regulated first and then maintained during the whole development of *C. sinensis* flower. According to the functional annotation, these DEGs were significantly enriched into the “gene expression”, “translation”, “nucleosome organization” and “protein folding”.

Cluster 3 contained 210 DEGs, whose expression were down-regulated first and then up-regulated during the whole development of *C. sinensis* flower, and significantly enriched into the GO term “phenylpropanoid biosynthetic process” and “L-phenylalanine metabolic process”.

Cluster 4 contained 51 DEGs, with expression maintained at first and then down-regulated during the whole development of *C. sinensis* flower. The “ATPase 11, plasma membrane-type” and “tubulin beta-5 chain” were highly enriched.

Cluster 5 contained 1034 DEGs, and their expression were maintained during the whole development of *C. sinensis* flower. Among them, 23 DEGs was significantly enriched in the GO term “protein phosphorylation”. Another significantly enriched GO term was “Endoplasmic Reticulum to Golgi Vesicle-Mediated Transport”.

There were 348 DEGs in Cluster 6, and the expression level of these DEGs remained unchanged and then increases during the whole development of *C. sinensis* flower. The most abundant GO term was “regulation of transcription, DNA-templated”.

Cluster 7 contained 496 DEGs, and their expression were firstly up-regulated and then down-regulated during the whole development of *C. sinensis* flower. The most abundant GO term was “intracellular signal transduction”, which included 3 “ras-related protein” and 1 “septum-promoting GTP-binding protein”.

Cluster 8 contained 877 DEGs, and their expression were firstly up-regulated and then maintained during the whole development of *C. sinensis* flower. Among them, the GO term “signaling” and “protein autophosphorylation” was significantly enriched.

Cluster 9 contained 274 DEGs, and their expression were continuously up-regulated during the whole development of *C. sinensis* flower. Among them, the GO term “glucan metabolic process” was significantly enriched.

Only a small number of genes, including UU and DD, were consistently expressed throughout the entire process of developing tea flowers, according to the research. While the expression levels of most genes were only altered during a specific stage of flower growth, which may be related to the development of the *C. sinensis* flower at a certain stage, these genes may be crucial to the entire process of *C. sinensis* flower development.

### Identification of the flower development-associated TF-encoding genes in DEGs

**Fig. 2:**
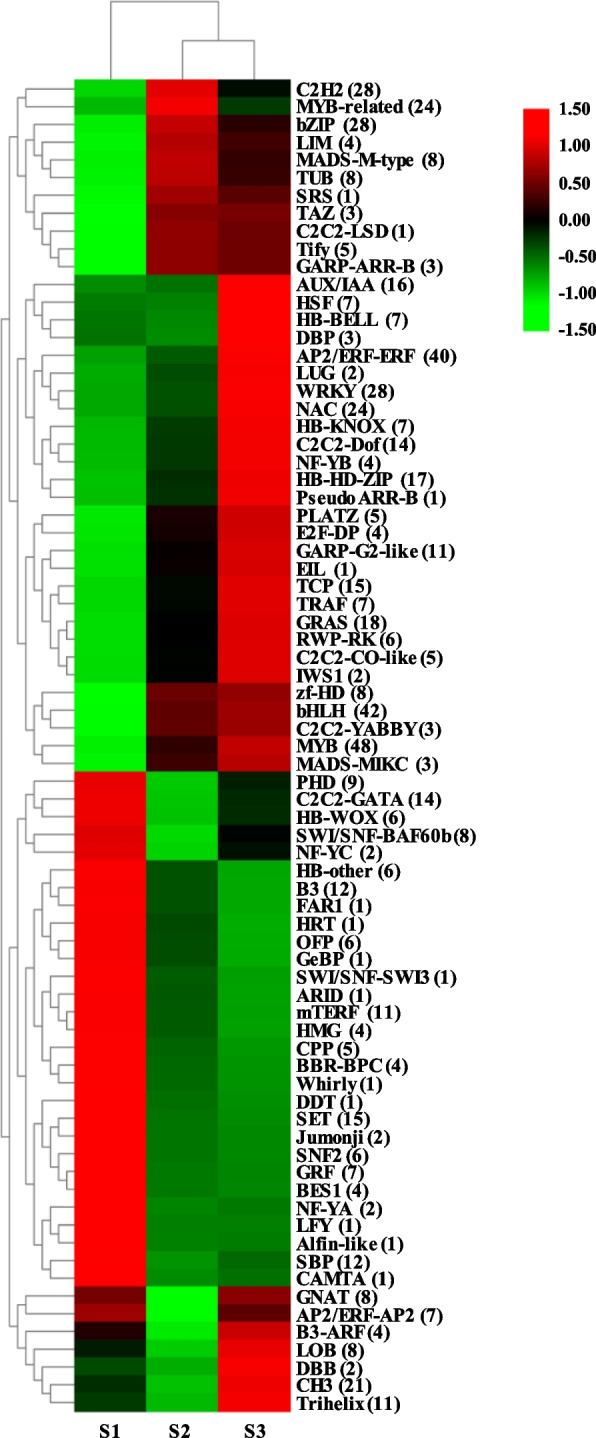
Clustering heatmap of overall expression trends of differentially expressed genes in flower development-related transcription factor families. The number of each transcription factor was showed in parentheses. S1-S3 represent the young bud stage, white bud stage and full bloom stage of *C. sinensis* flower developmental stages. The red-green schemes are labelled on the right side of heat map, and red to green represent high to low expression levels

From the 8611 DEGs shared by each pair of developmental phases of the three *C. sinensis* cultivars used in this work, 667 DEGs encoding transcription factors were found, which were then classified into 75 different transcription factor families (Fig. 2). The top 10 families with the largest number were *MYB* (48), *bHLH* (42), *AP2/ERF-ERF* (40), *WRKY* (28), *C2H2* (28), *bZIP* (28), *NAC* (24), *MYB*-related (24), *C3H* (21) and *GRAS* (18). In addition, besides *GRAS*, 16 *AUX/IAA* related to several hormones were also identified as DEGs. The results of the expression level analysis revealed that some TF families, including *C2C2-GATA*, *B3*, *SBP*, and other families, were considerably down-regulated after the S1 stage. In contrast, more TF families were significantly up-regulated with flower development, such as *MYB*, *bHLH*, *C2H2*, *GRAs*, *AUX/IAA* and *MADS-box*.

### Identification of flowering time-associated and flower development-related genes

**Fig. 3:**
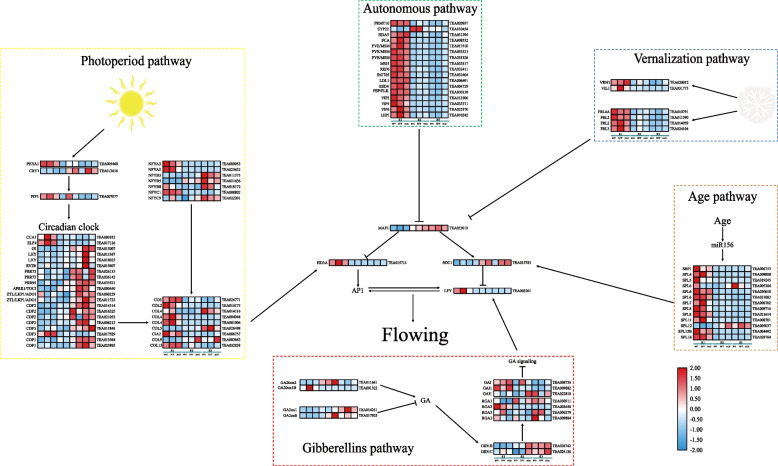
DEGs and putative schematic network of flowering induction pathways. The red-blue schemes are labelled on the right side of heat map, and red to blue represent high to low expression levels

This study screened 92 DEGs related to the flowering time and flower development from the 8611 DEGs shared by each pair of development stages of three cultivars by homology comparison with *A. thaliana* flower development related genes to identify the transcripts that may be related to the flowering time and flower development of tea. The majority of the genes involved with flowering time in *C. sinensis* could be categorized into five traditional flowering-related pathways (Fig. 3).

A total of 39 DEGs were identified in the photoperiod pathway, of which the transcript level of *PHYTOCHROME A 1* (*PHYA1*) and *PHYTOCHROME-INTERACTING FACTOR 3* (*PIF3*) showed a gradual decline with the development of *C. sinensis* flowers, while transcript level of *CRYPTOCHROME* 1 (*CRY1*) showed gradual upward trend. In addition, in the further circadian clock of photoperiod pathway, except for Japanese chestnut (*Castanea crenata* Sieb. et Zucc.) agglutinin 1 (*CCA1*), *EARLY FLOWERING 4* (*ELF4*) and *CYCLING DOF FACTOR 3* (*CDF3*) showed high expression in S1, other DEGs had the highest expression level in S3. Moreover, *CONSTANS* (*CO*) genes (*CO3*, *CONSTANS LIKE 1* (*COL1*), *COL2*, *COL4* and *CHLOROPLAST IMPORT APPARATUS 2* (*CIA2*) also showed high expression levels in S1 (Fig. 3). In autonomous pathway, vernalization pathway and age pathway, 13, 10 and 13 DEGs were identified, respectively. Most of these genes showed high expression levels in S1. However, the 13 DEGs involved in gibberellin pathway did not show regular regulation patterns.

The results of two-way ANOVA analysis showed that expression levels of all the 92 genes related to flowering time and flower development were significantly influenced by the development stage of the *C. sinensis* flower (Supplementary Table S5). Additionally, the higher F-values of the expression of *SPL7* in age pathway, *FLK* in autonomous pathway, *GID1C* and *GAI* in gibberellin pathway, and *RVE8*, *COL4* and *COL9* in photoperiod pathway were found under *C. sinensis* cultivar affect.

### Identification of DEGs involved in the pathways of various flowering-related hormones

#### Plant hormone content in C. sinensis flower

**Fig. 4:**
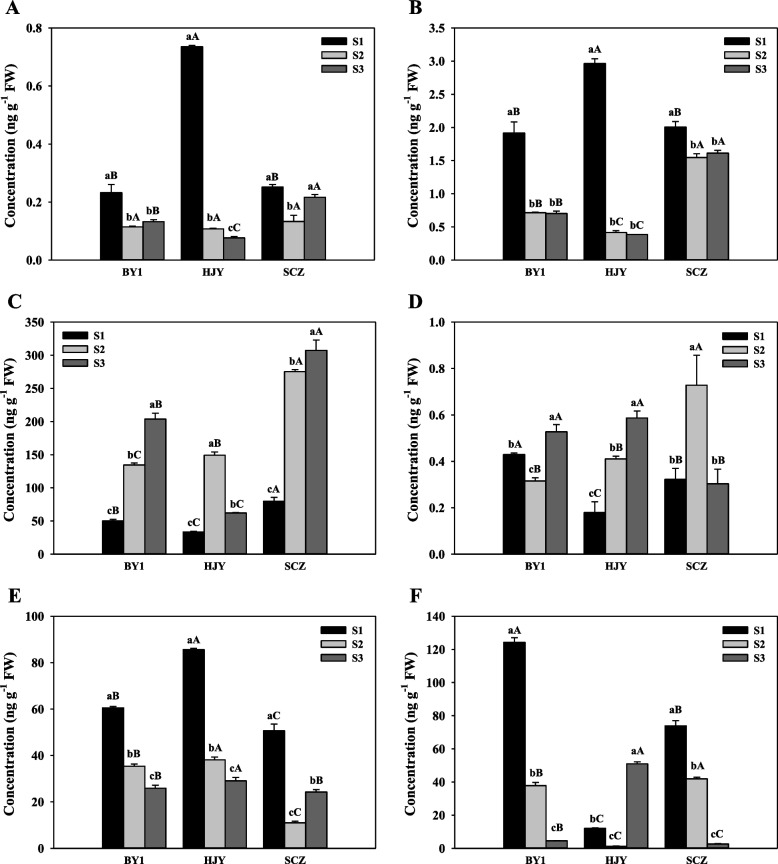
The content of plant hormone related to flower development. Indole-3-acetic acid (**A**), trans-zeatin riboside (**B**), abscisic acid (**C**), gibberellic acid 1 (**D**), salicylic acid (**E**), jasmonic acid. **F** BY1 represents the C. sinensis cv. ‘BaiYe1’, HJY represents the *C. sinensis* cv. ‘HuangJinYa’, SCZ represents the *C. sinensis* cv. ‘SuChaZao’, S1-S3 represent the young bud stage, white bud stage and full bloom stage of *C. sinensis* flower developmental stages. Different lowercase letters represent significant differences among different flower developmental stages of the same *C. sinensis* cultivar, and different uppercase letters represent significant differences among different *C. sinensis* cultivars at the same flower developmental stage

In order to explore the role of endogenous hormones in *C. sinensis* flower development, the contents of ABA, IAA, SA, JA, GA1, GA3, GA4 and TZR in *C. sinensis* flower of three cultivars at three developmental stages were determined (Fig. 4). Among them, the contents of IAA, TZR and SA in the three *C. sinensis* cultivars decreased significantly with the development of flowers (Figs. 4A, B and E). The ABA content of HJY increased firstly and then decreased, but the content of other cultivars increased significantly with the development of flowers (Fig. 4C). The contents of GA1 were high in all three stages of flower development (Fig. 4D), while GA3 was not detected in all stages of all cultivars, and the contents of GA4 were only detected in some stages, and the content were extremely low, which indicated that GA1 may be the dominant GAs in the flower development of *C. sinensis*. The contents of JA decreased significantly with the development of flowers, except HJY decreased at first and then increased (Fig. 4F).

#### Auxin biosynthesis and signal transduction pathway

**Fig. 5:**
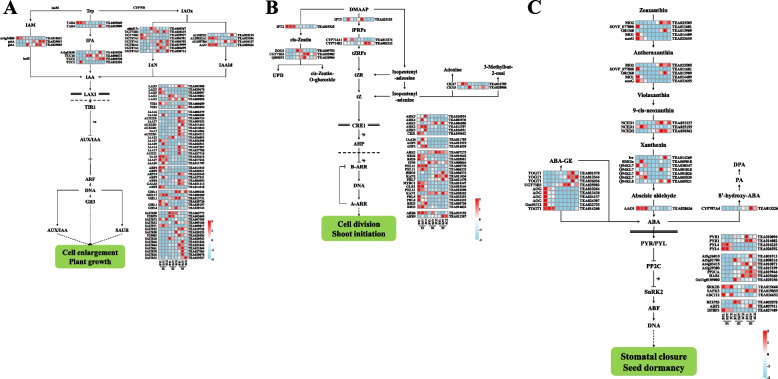
Differentially gene expression profiles of the auxin (**A**), cytokinin (**B**) and abscisic acid (**C**) biosynthesis and signaling transduction pathways. The red-blue schemes are labelled on the right side of heat map, and red to blue represent high to low expression levels

In the present study, 21 C-DEGs were found to be involved in auxin synthesis pathway, including 2 *TRYPTOPHAN AMINOTRANSFERASE OF ARABIDOPSIS 1* (*TAA1*), 4 *YUCCAs* (*YUCs*), 3 *ALDEHYDE DEHYDROGENASE* genes (*ALDHs*), 1 *ASCORBIC ACID OXIDASE* gene (*AAO*), 8 *UGT74B1* and 3 genes encoding amidase, among which *YUCs* were highly expressed in the S2 and S3 of *C. sinensis* flower development, while *AAO* was highly expressed in the early stage of *C. sinensis* flower development (Fig. 5A). In addition, 51 C-DEGs involved in auxin signal transduction were found, including 5 *AUX1* homologous genes, 2 *TIR1* homologous genes, 17 *AUX/IAAs*, 7 *ARFs*, 6 *GH3s* and 25 *SMALL-AUXIN-UP-RNAs* (*SAURs*), among which *SAURs* were highly expressed in the S2 and S3 of *C. sinensis* flower development.

The results of two-way ANOVA analysis showed that expression levels of all the 72 C-DEGs related to auxin biosynthesis and signal transduction were significantly influenced by the development stage of the *C. sinensis* flower (Supplementary Table S6). Additionally, the higher F-values of the expression of 2 genes (*ALDH7B4* and *UGT87A1*) related to the biosynthesis pathway from IAOx to IAAId to IAN were found under *C. sinensis* cultivar affect indicating their important role in the synthesis of IAA during *C. sinensis* flower development.

#### Cytokinin biosynthesis and signal transduction pathway

The results indicated that 9 C-DEGs involved in cytokinin synthesis pathway were found in this study, including 1 *ISOPENTENYL TRANSFERASE* gene (*IPT*), 2 *CYP735As*, 2 *CYTOKININ OXIDASE/DEHYDROGENASE* genes (*CKXs*), 1 *tRNA-DMATase*, and 3 *cZOGTs*, among which *cZOGTs* were highly expressed in the S2 and S3 of *C. sinensis* flower development (Fig. 5B). In addition, a total of 28 C-DEGs involved in cytokinin signal transduction were found, including 6 *CRE1s*, 3 *AHPs*, 17 *B-ARRs* and 2 *A-ARRs*, among which *GRE1s* and *AHPs* were mainly highly expressed in the S1 of *C. sinensis* flower development.

The results of two-way ANOVA analysis showed that expression levels of all the 37 C-DEGs related to cytokinin biosynthesis and signal transduction were significantly influenced by the development stage of the *C. sinensis* flower (Supplementary Table S7). Additionally, the higher F-value of the expression of 1 B-ARR gene, *HHO3*, was found under *C. sinensis* cultivar affect.

#### Abscisic acid biosynthesis and signal transduction pathway

The results indicated that 27 C-DEGs involved in abscisic acid synthesis pathway were found in this study, including 5 *ENZYME ZEAXANTHIN EPOXIDASE* genes (*ZEPs*), 3 *9-CIS-EPOXYCAROTENOID DIOXYGENASE* genes (*NCEDs*), 7 *SHORT-CHAIN DEHYDROGENASE/REDUCTASE-LIKE* (*SDRs*), 1 *AAO*, 1 *CYP707A* and 10 *AOGs*, among which most *ZEPs* and *AAO* were highly expressed in the S1 of flower development (Fig. 5C). In addition, 17 C-DEGs involved in abscisic acid signal transduction were found, including 4 *PYR/PYLs*, 7 *PP2Cs*, 3 *SnRK2s* and 3 *ABFs*, among which *PP2Cs* was highly expressed in the S3 of flower development, and the receptor genes *PYR1* (*TEA010094* and *TEA014082*) were highly expressed in the S2 and S3 of flower development and the receptor genes *PYL4* (*TEA016225* and *TEA026592*) were highly expressed in the S1 of flower development.

The results of two-way ANOVA analysis showed that expression levels of almost the 44 C-DEGs related to abscisic acid biosynthesis and signal transduction were significantly influenced by the development stage of the *C. sinensis* flower except the *Q94KL7* related to the biosynthesis pathway from xanthoxin to abscisic aldehyde (Supplementary Table S8).

#### Gibberellin biosynthesis and signal transduction pathway

**Fig. 6:**
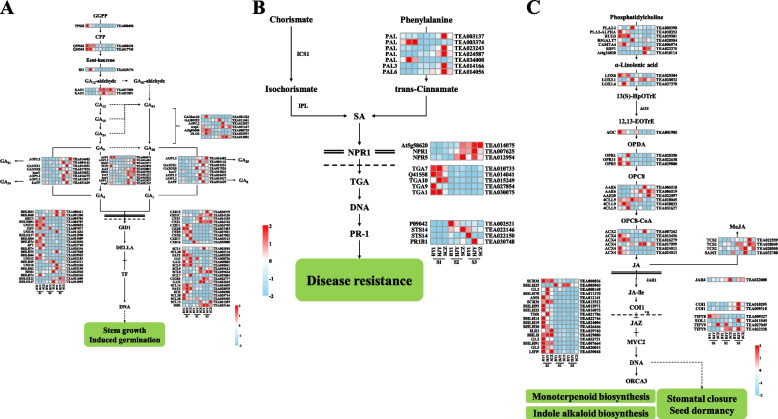
Differentially gene expression profiles of the gibberellin (**A**), salicylic acid (**B**) and jasmonic (**C**) biosynthesis and signaling transduction pathways. The red-blue schemes are labelled on the right side of heat map, and red to blue represent high to low expression levels

In the present study, 31 C-DEGs involved in gibberellin synthesis pathway were found, including 1 *COPALYL DIPHOSPHATE SYNTHASE* gene (*CPS)*, 2 *ENT-KAURENE SYNTHASE* genes (*KSs*), 1 *KAURENE OXIDASE* (*KO*), 2 *ENT-KAURENOIC ACID OXIDASE* (*KAOs*), 7 *GA20oxs*, 10 *GA3oxs* and 8 *GA2oxs*. among them, genes encoding *GA2oxs* were highly expressed in the S3 of *C. sinensis* flower development, while *CPSs*, *KSs* and *KO* were highly expressed in the S1 of *C. sinensis* flower development (Fig. 6A). In addition, 61 C-DEGs involved in gibberellin signal transduction were found, including 10 genes encoding *GID1* receptor protein, 18 genes encoding *DELLA* protein and 33 genes encoding *TF* (mainly *bHLH* family transcription factors), among which the genes encoding *DELLA* protein were highly expressed in the S1 of *C. sinensis* flower development.

The results of two-way ANOVA analysis showed that expression levels of almost the 92 C-DEGs related to gibberellin biosynthesis and signal transduction were significantly influenced by the development stage of the *C. sinensis* flower except for the DELLA encoding gene, *GAI1* (Supplementary Table S9). Additionally, the higher F-value of the expression of the *hxnY* related to the biosynthesis pathway from GA_9_ to GA_4_ and from GA_20_ to GA_1_, *EAPP* related to the biosynthesis pathway from GA_9_ to GA_51_, from GA_4_ to GA_34_, from GA_20_ to GA_29_ and from GA_1_ to GA_8_, 2 DELLA genes (*SCL9* and *SCL13*), and 1 TF gene (*BIM2*) were found under *C. sinensis* cultivar affect.

#### Salicylic acid biosynthesis and signal transduction pathway

The results showed that 7 C-DEGs encoding *PAL* were found to be involved in salicylic acid synthesis in phenylalanine ammonia-lyase pathway, and most of them were highly expressed in the S1 of flower development (Fig. 6B). In addition, 17 C-DEGs involved in salicylic acid signal transduction were found, including 3 *NONEXPRESSOR OF PATHOGENESIS-RELATED 1* (*NPR1s*), 5 *TGACG MOTIF-BINDING FACTOR* (*TGAs*) and 4 *PATHOGENESIS-RELATED 1* (*PR-1 s*), among which *NPR1s* and *PR-1 s* were highly expressed in the S2 and S3 of flower development, while *TGAs* were highly expressed in the S1 of flower development.

The results of two-way ANOVA analysis showed that expression levels of almost the 24 C-DEGs related to salicylic acid biosynthesis and signal transduction were significantly influenced by the development stage of the *C. sinensis* flower except for the PR-1 encoding gene, *TEA022150* (Supplementary Table S10).

#### Jasmonic acid biosynthesis and signal transduction pathway

In the present study, 30 C-DEGs involved in jasmonic acid synthesis pathway were found, including 2 *PHOSPHOLIPASE A2* (*PLA2s*), 4 *TGL4s*, 1 *DEFECTIVE IN ANTHER DEHISCENCE1* (*DAD1*), 3 *LIPOXYGENASE* (*LOXs*), 1 *ALLENE OXIDE CYCLASE* gene (*AOC)*, 3 *OXOPHYTODIENOIC ACID REDUCTASE* genes (*OPRs*), 6 *OPCL1s*, 6 *ACYL-COA OXIDASE* genes (*ACXs*) and 4 *JASMONIC ACID CARBOXYL METHYLTRANSFERASE* genes (*JMTs)*. Among them, *AOC* and *OPRs* were highly expressed in the S1 of flower development, while *JMTs* were highly expressed in the S2 and S3 of flower development (Fig. 6C). In addition, 25 C-DEGs involved in jasmonic acid signal transduction were found, including 1 *JAR1*, 2 *COI1s*, 4 *JAZs* and 18 *MYC2s*. Among them, *COI1s* was highly expressed in the S3 of flower development, while *MYC2s* was highly expressed in the S1 of flower development.

The results of two-way ANOVA analysis showed that expression levels of almost the 55 C-DEGs related to jasmonic acid biosynthesis and signal transduction were significantly influenced by the development stage of the *C. sinensis* flower except for the JAZ encoding gene, *TEA027049* (Supplementary Table S11). Additionally, the higher F-value of the expression of the 2 genes (*TEA000390* and *TEA028584*) related to the biosynthesis pathway from phosphatidylcholine to α-linolenic acid, 1 gene (*TEA020350*) related to the biosynthesis pathway from OPC8 to OPC8-CoA, 1 gene (*TEA028049*) related to the biosynthesis pathway from JA to MeJA were found under *C. sinensis* cultivar affect.

#### Verification of RNA-Seq results by RT-qPCR

**Fig. 7:**
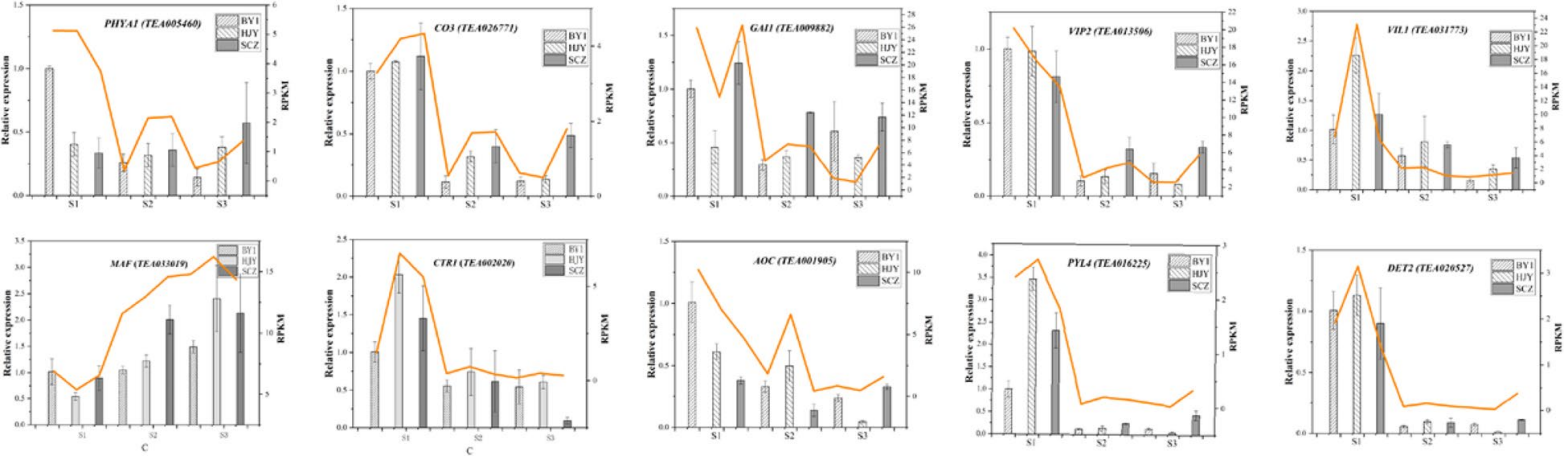
Quantitative real-time PCR validation of 10 DEGs

To verify the gene expression profile in our RNA-Seq results in this study, the expression levels of 10 random DEGs were verified using RT-qPCR. As shown in Fig. 7, the relative expression level of the selected RNA showed a consistent trend with the sequencing results, which proved the accuracy of sequencing data.

## Discussion

### Genome-wide identification of flower development in C. sinensis

Flowering is an important process in the transition from vegetative growth to reproductive growth in the life cycle of plants [24]. Although studies on *C. sinensis* flowers have grown steadily in recent years, there has been little research on the intricate network that controls flower development in tea plants. Furthermore, these studies have all focused on a single type of tea plants, with no comparisons between them [3, 25]. The current study focused on the differentially expressed genes related to *C. sinensis* flower formation by sequencing the transcriptomes of the flowers of three *C. sinensis* cultivars at three developmental stages, which provided a reference for further analysis of the flowering mechanism of *C. sinensis*.

The expression of the majority of genes was repressed during the growth of the *C. sinensis* flower, as evidenced by the large rise in the number of genes with very low expression (RPKM < 1) in the three cultivars of *C. sinensis* flowers after S1. Additionally, the findings of the association between developmental stages and the number of DEGs revealed that in all cultivars, the differences between the S1 and S3 stages were large, while those between the S2 and S3 stages were relatively moderate. Furthermore, we defined the core transcriptome of *C. sinensis* development by screening for DEGs shared by the three *C. sinensis* cultivars between each developmental stage. The results of cluster analysis of core transcriptome of *C. sinensis* development showed that 5355 DEGs were clustered into 9 categories. Among them, only a few genes were continuously expressed throughout the whole process of *C. sinensis* flower development, such as UU and DD. The genes in the UU group may play important roles in flower development which showed that several *XYLOGLUCAN ENDOTRANSGLYCOSYLASE/HYDROLASE* genes (*XTHs*) were found to be significantly enriched (Fig. 1D). Cell wall remodeling frequently occurs concurrently with cell growth and proliferation during plant growth and development. *XTHs*, which are extensively distributed in many plant tissues and cells, encode the essential enzyme in the process of modifying plant cell walls. It can aid in the creation and breakdown of cell walls, as well as relax and reinforce cell walls [26, 27]. Additionally, *XTHs* are crucial for the growth and development of flowers. By combining study of proteomic and transcriptome data for orchid labial and medial petals, Li et al. revealed that *XTHs* might encourage the development of orchid (*Cymbidium* spp.) petal morphology [28]. The DD group includes some genes enriched on RNA polymerase transferases, mitochondrial import inner membrane translocases, nuclear pore complex proteins, and homeobox protein *ATH1*, however, these genes had low expression levels and might play a secondary role in flower development.

TFs are crucial for a variety of processes related to plant growth and development [29]. In the present study, a large number of TF families related to flower development were identified, among which the genes in *MYB*, *bHLH*, *AP2/ERF-ERF*, *bZIP*, *MYB*-related, *C2H2*, *MADS-box* and *AUX/IAA* families were continuously up-regulated during flower development indicating that these genes families were likely to be involved in the regulation of *C. sinensis* flower formation (Fig. 2). These gene families have also been shown to be involved in the development of rice [30] and apple [31] flower. *MYB* family genes contain DNA-binding domains, some of which have been identified as regulators of floral development [32]. As expected, 48 *MYB* family genes were differentially expressed in this study. In addition, the *MYB* TF can promote the growth of *A. thaliana* petals and stamens. For example, *MYB21* exerts a negative feedback on the expression of JA biosynthesis-related genes by reducing JA levels, thereby controlling flower development [33]. *bHLH* is the TF family with the second largest number of differentially expressed genes, and it can regulate various processes related to flower development [34]. It has been reported that the morphogenesis of floral organs is controlled by multiple *MADS-box* genes, and the loss of any essential *MADS-box* gene function may lead to homologous transformation of floral organs. For instance, MADS-box proteins of the SEPALLATA subfamily are involved in the formation of flower meristems in spikelet in rice [35]. Additionally, 4 *MADS-box* genes similar to *AGL9* play a role in flower formation and transformation in *Arabidopsis* [36]. In this study, a total of 11 genes belonged *MADS-box* family were identified, of which 8 were *M-type-MADS* (which encode 5 *AGL*s, 1 *SOC1*, and 2 hypothetical proteins, respectively) and 3 were *MIKC-MADS* (1 unknown protein, 1 growth regulator and 1 *MADS-box* transcription factor 23). *AGL* is an E-type functional gene for the development of floral organs, and it participates in the entire process of floral organ formation [36], and *SOC1* is an integrated gene in the flower induction pathway, which determines the flowering time of plants [12]. *MADS-box* transcription factor forms a classic ‘ABCDE’ flower development model, which is involved in the development of floral organs and the determination of reproductive meristem attributes [37, 38]. In the ‘ABCDE’ flower development model, the ‘E-class’ function is responsible for regulating the formation of four-wheeled flower organs, and it is an important functional gene in *MADS-box* transcription factors [39].

A total of 92 DEGs related to flowering time and flower development were found in the core transcriptome of *C. sinensis* by homology comparison with the reported *A. thaliana* related genes. The photoperiod pathway, autonomous pathway, vernalization pathway, hormone pathway and age pathway are essential pathways in the flowering process of plants, and these pathways can form a complex genetic network to further regulate the flowering process of plants. The genes participating in these pathways in this complex genetic network are controlled by numerous flowering time integrators, which combine signals from many pathways to regulate flowering as opposed to acting individually and directly to govern plant flowering. According to the floral regulatory network existing in the model plant *A. thaliana*, the possible regulatory network in the flowering process of *C. sinensis* was mapped (Fig. 3). In the photoperiod pathway, the *PHYA* gene that senses far-red light was highly expressed in S1, and the *CRY1* gene that senses blue light was highly expressed in S2. This might be because different developmental stages of *C. sinensis* have different requirements for light quality. Most of the circadian clock genes were highly expressed in the later stages of flower development, and through complex interactions, these genes then transmit the flowering signal to the downstream *CO*. *CO* is an important flower gene in the photoperiod pathway [40], which was highly expressed in S1 of flower development and induced flower formation by acting on the *HD3A* gene in the present study, indicating that the photoperiodic pathway promotes the process of *C. sinensis* flower formation. In the autonomous pathway, genes such as *PRMT10*, *SYP22*, *HDA5*, *FCA*, FVE/*MSI*, *MSI5*, *REF6* and *JMJ705* were highly expressed in the early stage, and indirectly promoted the expression of *SOC1* by inhibiting the expression of *MAF1*, thereby inducing flowering in *C. sinensis*. By encouraging the early expression of genes like *VRN1* and *VIL1* and indirectly encouraging the expression of *SOC1* by suppressing the expression of *MAF1*, low temperature stimulates *C. sinensis* flowering in the vernalization pathway. In the gibberellin pathway, the gibberellin synthesis gene *GA20OX* is highly expressed in the early stage of flower development and promotes the synthesis of gibberellin. The expression level of gibberellin receptor gene, *GID1*, was low in the early stage of flower development, which reduces the inhibition of the expression of DELLA protein and promotes *C. sinensis* flowering. In the age pathway, the *miR156* promotes *C. sinensis* flowering by promoting the expression of the downstream gene, *SPL* [41]. The expression of flower meristem characteristic gene *LFY* was the highest in the early flowering period, indicating that the expression of *LFY* increased rapidly before flowering.

*SOC1*, a member of *MADS-box* family, participates in a series of life activities, such as organ development, flowering and dormancy. *SOC1* integrates various flowering regulation pathways such as autonomy, vernalization and age pathway, and can positively regulate flower development genes [42]. Moreover, *FLC* is the central gene of vernalization pathway, which negatively regulates flowering by inhibiting the expression of *FT* and *SOC1* in *A. thaliana* [43, 44]. However, none of these three *C. sinensis* genomes found genes homologous to *A. thaliana FLC* gene. Similarly, Liu et al. reported that no gene homologous to the *A. thaliana FLC* gene was found in the *C. sinensis var. sinensis* genome annotation, but was homologous to that of grape (*Vitis vinifera*), indicating that *FLC* might be specific to *Brassica* species [3]. Therefore, the function of *FLC* gene in *C. sinensis* needs more research. Furthermore, according to “ABCDE model”, *LFY* plays a key role in flower bud development by regulating the expression of organ identity genes, and *LFY* is an FM identity gene [45, 46].

### Endogenous hormone synthesis and signal transduction affect flowering time and flower development in C. sinensis

Phytohormones, as important components in regulating plant flowering time and flower development, have been confirmed in model plants [47]. However, the complex hormonal regulatory network of perennial flower development remains unclear. In this study, we revealed the changes in the content of various plant endogenous hormones and the expression of related genes during the development of *C. sinensis*, which will help to understand the role of plant endogenous hormones in the flowering of woody plants.

The role of auxin in plant flowering transition has been extensively studied in model plants, but little is known about its function in woody plants. Endogenous auxin has been reported to gradually concentrate in the SAM during the flowering transition in plants, suggesting that auxin plays a key role in mediating the flowering transition in strawberries [48]. The IAA content in the present study was higher in the S1, indicating that auxin may be involved in the flowering induction of *C. sinensis*, which was similar to Guo’s report on seasonal roses [49]. In addition, auxin content and *TAR*, *AAO*, *UGT74B1* and other genes were down-regulated synchronously from S1 to S2, while the expression of *ALDH* and *YUC* were up-regulated, indicating that auxin-related genes were involved in the floral transition process of *C. sinensis*. As a typical auxin-responsive family, *ARF* is involved in the transformation and development of *C. sinensis* and *ARF3* has been shown to function to integrate *AGAMOUS* and *APETALA2* during floral meristem formation in *A. thaliana* [50].

Although the function of CK in flower development is not entirely understood, it is known that it regulates cell division and differentiation in floral meristems [51]. The degradation of CK is very important in its homeostasis. Studies have shown that cytokinin oxidase/dehydrogenase 3 (*CKX* 3) and *CKX5* are involved in the regulatory activity of *A. thaliana* reproductive meristem [52]. A *CKX5* gene was found in the present study which has high expression levels in S2 and S3. However, *CKX7* has a concentrated high expression level in S1 of three cultivars. We speculate that *CKX7* and *CKX5* play different roles in the process of CK participating in *C. sinensis* flower development, that is, slow down the degradation of CK in the middle and late stage of flower development, thus accelerating the division and differentiation of flower organ cells.

ABA is a sesquiterpene plant hormone involved in the growth and development process, including the synthesis of seed storage proteins and lipids, dehydration tolerance of seeds, dormancy and flowering of seeds [53]. In the present study, the ABA contents were higher than other hormones, and were significantly higher in S2 and S3 than that in S1 (Fig. 4C). The expression levels of ABA synthesis gene, *NCED*, and receptor gene, *PYR*, gradually increased during the transition to flower formation, indicating that ABA signal transduction-related genes might play a role in promoting flowering in *C. sinensis*. Similar result has been reported by Cui et al. [54], that ABA can promote the expression of lychee *LcAP1* and trigger flower development. However, ABA played the opposite role in rose development [49]. In ABA signal transduction, *ABA HYPERSENSITIVE 1* (*HAB1*) is involved in flowering control [55]. It is a phosphatase type 2Cs (PP2C), a negative regulator of ABA signal, and has been found to induce flowering in *A. thaliana* [55, 56]. Additionally, *SnRK1*, a key component of the ABA signaling pathway, is involved in glucose metabolism, and plays an active regulatory role in ABA signaling [54], suggesting that sugar may interact with ABA to mediate flowering transition. FCA is an ABA-binding protein, and the application of ABA affects the ratio of long and short spliced forms of FCA, thereby inhibiting flowering [57].

GA is a crucial hormone that affects plant development, flowering induction, and seed germination [50]. In the present study, the contents of GA_1_ were high in all sample, but GA_3_ was not detected in all sample species, and the content of GA_4_ was only detected in some samples with extremely low value, which indicated that GA_1_ might play a major role in the development of *C. sinensis*. In addition, the contents of GA_1_ in both S2 or S3 were significantly higher than that in S1, and GA biosynthesis genes (such as *KAO*, *GA20ox*, *GA2ox*, and *hxnY*, etc.) had higher expression levels in both S2 and S3 (Fig. 4D and Fig. 6A). GA1 is the main bioactive form in plants, which exists in different tissues and controls different development processes, such as seed germination, stem elongation, leaf growth and flower development [58]. In addition, it has been reported that GAs is closely related to *A. thaliana* DELLA proteins GAI (GA-insensitive), RGA (suppressor of ga1-3), RGL1 (RGA-like 1), RGL2 and RGL3, and they are the key transcription regulatory factors that inhibit GA response [59]. GAs regulates the development and fertility of flowers by inhibiting the function of DELLA protein [60]. The expression levels of most of the DELLA genes screened in this study continued to decline with the developmental stage of *C. sinensis* flower (Fig. 6A) which indicated that DELLA played a negative regulatory role in the development of *C. sinensis* flower.

SA is a member of a category of phenolic compounds that have aromatic rings and hydroxyl groups or their functional groups [61]. PAL controls SA synthesis and is involved in the mechanism that controls stress-induced *Pharbitis* flowering [62]. Exogenous SA might reverse the phenotype of mutant *co-1* in the late flowering stage, according to genetic analysis of the genes involved in SA interaction throughout photoperiod. Additionally, this demonstrates that SA controls blooming via a CO-dependent mechanism that interacts with a photoperiod-dependent pathway [63]. In addition, in the process of SA signal transduction, 3 *NPR1* genes and 4 *PR-1* genes were found to have high expression in the middle and late stage of *C. sinensis* flower development, while 5 *TGA* genes only had high expression in S1 (Fig. 6B). It has been reported that *NPR1* can't directly combine with *PR-1* promoter, but is recruited to the promoter through physical interaction with *TGAs*, thus regulating the expression of *PR-1* [64, 65]. *NPR1* plays a crucial role in SA regulation of the plant defense response as a transcriptional co-activator [66]. Therefore, we hypothesize that SA can interact with *NPR* to limit the function of *TGA* before up-regulating *PR-1* expression to encourage tea flowering. However, *NPR3*, a *NPR1*-like gene, has been proved that its expression level will decrease with the flowering of *A. thaliana*, and is confirmed as the inhibitor of *NPR1*-dependent and independent pathway [67]. Therefore, the role of *NPR1* in plant flower development cannot be ignored, and *NPR3* may also inhibit plant flowering by negatively regulating the functions of other *NPR1*. Furthermore, *TGA1* and *TGA4* were expressed around the border of flower organs in *A. thaliana*, which was necessary for inflorescence structure, meristem maintenance and flowering [68], while *A. thaliana* without *TGA7* showed a delayed flowering phenotype [69]. Additionally, it has been suggested that overexpression of CruTGA4 (*Capsella rubella*) in *A. thaliana* may limit FT expression by interacting with *CO*, delaying *A. thaliana* flowering [70]. These findings seem to suggest that *TGAs* will play a variety of roles in controlling plant flower development. *TGAs*, however, only displayed high transcription levels in S1, showing that they are negatively regulated in the growth of the *C. sinensis* flower.

Jasmonic acid and its derivatives (named jasmonate ester) are oxygenated derivatives of lipid plant hormones, linoleic acid and linolenic acid [71]. Numerous developmental processes, including the storage of nitrogen, fruit ripening, senescence, and blooming, are mediated by JA and jasmonate molecules [72]. Jasmonic acid synthesis and accumulation have been identified as the essential steps in flower development in plant breeding. Numerous studies demonstrate that *A. thaliana* contains mutations that cause long-term male sterility in perception (coi1) and JA synthesis (*FAD378*, *DAD1*, *AOS*, *OPR3* and *ACX1/5*) [73, 74]. In the present study, 2 *COI1* genes were identified with high expression levels in S3. The high expression of *COI1* in the later stage of *C. sinensis* flower development ensures the normal development of flower organs. It is reported that transcription factors *MYC2/3/4/5* and *MYB21/24* of *A. thaliana R2R3-MYB* are induced and protected by *JAZ* inhibitor, and are identified as regulatory factors controlling stamen development [33, 75]. *JAZs* interact with *MYB21* and *MYB24* to weaken their transcription function. After receiving a JA signal, *COI1* attaches *JAZs* to the SCF^COI1^ complex, thereafter ubiquitination and 26S proteasome degradation release *MYB21* and *MYB24* to stimulate the expression of many genes. These genes are crucial for JA since they control anthrax growth and filament elongation [33].

**Fig. 8:**
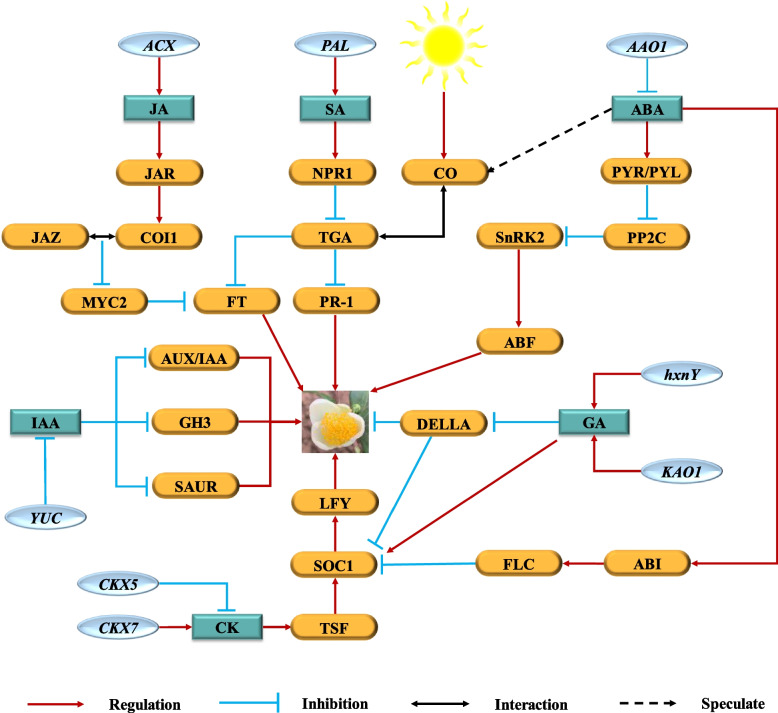
Multiple hormones regulating flowering time

To sum up, according to the results of this study, based on the comprehensive study of flowering of other plants, we deduced a complex hormone regulation network of *C. sinensis* flowering (Fig. 8), which connected independent hormone signals in series through functional proteins. As an important integrator of plant flowering signal, FT integrates the signal transduction of JA, ABA and SA in *C. sinensis*: 1) in the signal transduction process of JA, the interaction between *COI1* and *JAZ* weakens the transcription function of *MYC2*, and *MYC* further inhibits the function of FT [76]; 2) in the signal transduction process of SA, *TGA* may inhibit the expression of FT by interacting with *CO*, an important regulatory factor in photoperiod pathway [70]; 3) it has been reported that ABA signal may be involved in activating *CO* transcription or enhancing the function of CO protein or regulating *MYCs* transcription factor, thereby inhibiting the expression of *FT* [77, 78]. Moreover, *SOC1* also integrates the signal transduction of ABA, GA and CK in promoting flower development: 1) ABA can up-regulate the expression of transcription factor *ABI* to activate *FLC* transcription, reduce the expression of *SOC1* and delay the flowering of plants [79]; 2) GA signal can directly up-regulate the expression of activation factor *SOC1* of *LFY*, which is independent of DELLA [80]; 3) CK can activate the collateral homologous of FT, *TWIN SISTER OF FT* (*TSF*), and downstream *SOC1*, and then activate *LFY* to promote flower development [81]. Therefore, we predict that *MYC*, *FT*, *SOC1* and *LFY* play a key role in the process of plant endogenous hormones regulating *C. sinensis* flower development. The results of this study can provide reference for the further study of *C. sinensis* flowering mechanism. Therefore, more research is needed to reveal the exact function of the core transcriptome of flower development to clarify the final flowering mechanism of *C. sinensis*.

## Conclusions

In this study, a 92-genes’ *C. sinensis* flower development core transcriptome with 4 flowering time integrators (1 *HD3A*, 1 *SOC1* and 1 *LFY*, and *SOC1*) were identified from the transcriptome of three *C. sinensis* cultivars. In addition, we screened out 217 differentially expressed genes related to plant hormone synthesis and 199 differentially expressed genes related to plant hormone signal transduction in *C. sinensis* flower development stage. And we speculated that *MYC*, *FT*, *SOC1* and *LFY* played the key role in the process of plant endogenous hormones regulating *C. sinensis* flower development by constructing a complex hormone regulation network of *C. sinensis* flowering.

## Materials and methods

### Plant materials

Three 8-year-old *C. sinensis* cultivars, ‘SuChaZao’ (SCZ), ‘HuangJinYa’ (HJY), and ‘BaiYe1’ (BY1) used in this study were grown in the same tea plantation at the Tea Research Institute of Tianmu Lake in Liyang, Changzhou, Jiangsu, China (31°20' N, 119°23' E) with normal pest management and fertilizer application. Flower samples of three development stages young bud stage (S1), white bud stage (S2) and full bloom stage (S3) were harvested separately from three *C. sinensis* cultivars on the same day (Supplementary Fig. S2). For each flower sample, three biological replicates were performed. Each biological replicate consisted of at least 10 floral buds randomly collected from five tea trees of each variety block. All samples were frozen in liquid nitrogen immediately after harvesting, and stored at -80 °C for RNA extraction and phytohormone quantification.

### RNA extraction, cDNA library construction and sequencing

Total RNA was obtained from each frozen sample in liquid nitrogen using TRIzol (Invitrogen, Carlsbad, CA, USA) according to the manufacturer’s protocol. The methods of the quality and integrity of total RNA and the constructing and sequencing cDNA library refer to Wang et al. [82].

### Fundamental and annotation analysis

Based on the HISAT2 alignment BAM file, feature Counts v1.6.2 [83] was used to estimate and quantify gene expression with default parameters, yielding raw read count for each RNA genes. Gene expression was normalized by reads per kilobase of exon per million reads mapped (RPKM). The data were processed according to the method of [84], that is, all the genes in all samples were merged into a non-redundant list by using Cuffmerge, the gene expression of the samples with three biological repetitions was merged (taking the average value of RPKM) and the genes with RPKM less than 1 in all samples were eliminated, and the remaining 22,927 genes were used for subsequent analysis. Differential expression genes (DEGs) were identified with false discovery rate (FDR) ≤ 0.05 and log_2_ |fold change|≥ 1 in each pairwise comparison using edgeR package [85]. Cluster analysis was carried out according to Yu et al. [86]. In brief, by comparing the expression trends of each gene in two successive adjacent development stages (‘S1 vs. S2’ and ‘S2 vs, S3’), it is considered that the gene was up-regulated in the development process based on the standard of fold change ≥ 2, which was classified as “up”. With fold change ≤ 0.5 as the standard, it was considered that the gene was down-regulated during development and classified as “down”. Genes with fold change between 0.5 and 2 were regarded as not regulated and classified as “maintain”. According to the comparison between the two groups at three developmental stages, the gene could be divided into 9 clusters, namely, down-down (DD), down-maintain (DM), down-up (DU), maintain-down (MD), maintain-maintain (MM), maintain-up (MU), up-down (UD), up-maintain (UM) and up-up (UU).

Then Gene Ontology (GO) and Kyoto Encyclopedia of Genes and Genomics (KEGG) pathway analyses were performed according to previous reports [87–89].

### Quantification of phytohormones

The indole-3-acetic acid (IAA), gibberellins (GAs), trans-zeatin riboside (TZR), abscisic acid (ABA), salicylic acid (SA) and jasmonic acid (JA) were measured at three flower developmental stages of each tea variety. About 0.5 g (fresh weight) of each plant part was subjected to analysis. Quantification of phytohormones was performed as described previously [90] using high-performance liquid chromatography coupled with a tandem quadrupole mass spectrometer equipped with an electrospray interface (HPLC–ESI–MS/MS). The specific operating parameters were shown in Supplementary Table S2 and S3.

### Statistical analysis

Data analysis and correlation analysis were performed using SPSS software (SPSS Inc. version 22.0, IL, Chicago, USA, 2013) under Duncan’s test. The data diagrams were drawn with SigmaPlot software (SigmaPlot, version 12.5).

## Supplementary information

**Additional file 1: Fig. S1:** The ABCDE model and the quartet model of floral organ development in *Arabidopsis* *thaliana.*

**Additional file 2: Fig. S2:** Morphological characteristics of flower organs at three flower developmental stages in three *Camellia* *sinensis* varieties. BY1 represents the* C. sinensis cv. *‘BaiYe 1’, HJY represents the* C. sinensis cv. *‘HuangJinYa’, SCZ represents the* C. sinensis cv. *‘SuChaZao’, S1-S3 represent the three flower developmental stages.

**Additional file 3: Fig. S3:** Gene expression analysis of the three *C. sinensis* varieties during flower development. For each FPKM interval, the average number of expressed genes and the corresponding SD values are shown in a white box. "*" and "**" indicate significant differences between the last developmental stages at the level of 0.05 and 0.01, respectively. BY1 represents the* C. sinensis cv. *‘BaiYe 1’, HJY represents the* C. sinensis cv. *‘HuangJinYa’, SCZ represents the* C. sinensis cv. *‘SuChaZao’, S1-S3 represent the three flower developmental stages.

**Additional file 4: Table S1:** The primers used for qRT-PCR verification.

**Additional file 5: Table S2:** ESI-MS/MS parameters for determination of eight phytohormones.

**Additional file 6: Table S3:** The parameters of gradient elution of HPLC.

**Additional file 7: Table S4:** Summary of RNA-Seq data and mapping metrics.

**Additional file 8: Table S5:** Two-way ANOVA test *F*-value of expression levels of the genes related to flowering time and flower development for three *C. sinensis* cultivars (BY1, HJY and SCZ) affected by development stage (S) and cultivar (C).

**Additional file 9: Table S6:** Two-way ANOVA test *F*-value of genes expression levels related to auxin biosynthesis and signaling transduction for three *C. sinensis* cultivars (BY1, HJY and SCZ) affected by development stage (S) and cultivar (C).

**Additional file 10: Table S7:** Two-way ANOVA test *F*-value of genes expression levels related to cytokinin biosynthesis and signaling transduction for three *C. sinensis* cultivars (BY1, HJY and SCZ) affected by development stage (S) and cultivar (C).

**Additional file 11: Table S8: **Two-way ANOVA test *F*-value of genes expression levels related to abscisic acid biosynthesis and signaling transduction for three *C. sinensis* cultivars (BY1, HJY and SCZ) affected by development stage (S) and cultivar (C).

**Additional file 12: Table S9: **Two-way ANOVA test *F*-value of genes expression levels related to gibberellin biosynthesis and signaling transduction for three *C. sinensis* cultivars (BY1, HJY and SCZ) affected by development stage (S) and cultivar (C).

**Additional file 13: Table S10: **Two-way ANOVA test *F*-value of genes expression levels related to salicylic acid biosynthesis and signaling transduction for three *C. sinensis* cultivars (BY1, HJY and SCZ) affected by development stage (S) and cultivar (C).

**Additional file 14: Table S11: **Two-way ANOVA test *F*-value of genes expression levels related to jasmonic biosynthesis and signaling transduction for three *C. sinensis* cultivars (BY1, HJY and SCZ) affected by development stage (S) and cultivar (C).

## Data Availability

All data generated or analyzed during this study are included in this published article [and its supplementary information files].

## Abbreviations

AAO: Ascorbic Acid Oxidase
A-ARR: Type-A Two-Component Response Regulator
ABA: Abscisic Acid
ACX: Acyl-Coa Oxidase
ALDH: Aldehyde Dehydrogenase
AOC: Allene Oxide Cyclase
AP1: Apetala1
ARF: Auxin Response Factor
B-ARR: Type-B Two-Component Response Regulator
BY1: *C. sinensis* cv ‘BaiYe1’
CCA1: Japanese Chestnut (*Castanea crenata* Sieb. et Zucc.) agglutinin 1
CDF3: Cycling Dof Factor 3
CIA2: Chloroplast Import Apparatus 2
CK: Cytokinins
CO: Constans
COL1: Constans 1
CPS: Copalyl Diphosphate Synthase
CRY1: Cryptochrome 1
DD: Down-Down
DEG: Differential Expression Gene
DM: Down-Maintain
DU: Down-Up
ELF4: Early Flowering 4
EMF: Embryonic Flower
FDR: False Discovery Rate
FLC: Flowering Locus C
FM: Floral Meristem
FT: Flowering Locus T
Gas: Gibberellins
GO: Gene Ontology
HJY: *C. sinensis* cv ‘HuangJinYa’
IAA: Indole-3-Acetic Acid
IM: Inflorescence Meristem
JA: Jasmonic Acid
JMT: Jasmonic Acid Carboxyl Methyltransferase
KAO: Ent-Kaurenoic Acid Oxidase
KEGG: Kyoto Encyclopedia of Genes and Genomics
KO: Kaurene Oxidase
LFY: Leafy
LOX: Lipoxygenase
MD: Maintain-Down
MM: Maintain-Maintain
MU: Maintain-Up
NCED: 9-Cis-Epoxycarotenoid Dioxygenase
NPR: Nonexpressor of Pathogenesis-Related
OPR: Oxophytodienoic Acid Reductase
PHYA1: Phytochrome A 1
PIF3: Phytochrome-Interacting Factor 3
PLA2: Phospholipase A2
PR-1: Pathogenesis-Related 1
RPKM: Per Million Reads Mapped
S1: Young Bud Stage
S2: White Bud Stage
S3: Full Bloom Stage
SA: Salicylic Acid
SAM: Shoot Apical Meristem
SAUR: Small-Auxin-Up-Rna
SCZ: *C. sinensis* cv ‘SuChaZao’
SDR: Short-Chain Dehydrogenase/Reductase-Like
SOC1: Suppressor of Overexpressionof Constans 1
TAA: Tryptophan Aminotransferase of Arabidopsis
TFL: Terminal Flower
TGA: Tgacg Motif-Binding Factor
TZR: Trans-Zeatin Riboside
UD: Up-Down
UM: Up-Maintain
UU: Up-Up
YUC: Yucca
ZEP: Enzyme Zeaxanthin Epoxidase
