# Supplementary material for: Transcriptome analysis reveals the roles of phytohormone signaling in tea plant (Camellia sinensis L.) flower development
Source: BMC Plant Biol. 2022 Oct 4;22:471. doi: 10.1186/s12870-022-03853-w (PMC9531472; doi:10.1186/s12870-022-03853-w)
Supplement: Supplementary file 1 — Additional file 1: Fig. S1: The ABCDE model and the quartet model of floral organ development in Arabidopsis thaliana. [file 12870_2022_3853_MOESM1_ESM.docx]

**Supplementary Fig. S1** The ABCDE model and the quartet model of floral organ development in *Arabidopsis* *thaliana.*
