# Supplementary material for: Transcriptome analysis reveals the roles of phytohormone signaling in tea plant (Camellia sinensis L.) flower development
Source: BMC Plant Biol. 2022 Oct 4;22:471. doi: 10.1186/s12870-022-03853-w (PMC9531472; doi:10.1186/s12870-022-03853-w)
Supplement: Supplementary file 2 — Additional file 2: Fig. S2: Morphological characteristics of flower organs at three flower developmental stages in three Camellia sinensis varieties. BY1 represents the C. sinensis cv. ‘BaiYe 1’, HJY represents the C. sinensis cv. ‘HuangJinYa’, SCZ represents the C. sinensis cv. ‘SuChaZao’, S1-S3 represent the three flower developmental stages. [file 12870_2022_3853_MOESM2_ESM.docx]

**Supplementary Fig. S2** Morphological characteristics of flower organs at three flower developmental stages in three *Camellia* *sinensis* varieties. BY1 represents the *C. sinensis cv.* ‘Baiye 1’, HJY represents the *C. sinensis cv.* ‘Huangjinya’, SCZ represents the *C. sinensis cv.* ‘Suchazao’, S1-S3 represent the three flower developmental stages.
