# Supplementary material for: Transcriptome analysis reveals the roles of phytohormone signaling in tea plant (Camellia sinensis L.) flower development
Source: BMC Plant Biol. 2022 Oct 4;22:471. doi: 10.1186/s12870-022-03853-w (PMC9531472; doi:10.1186/s12870-022-03853-w)
Supplement: Supplementary file 3 — Additional file 3: Fig. S3: Gene expression analysis of the three C. sinensis varieties during flower development. For each FPKM interval, the average number of expressed genes and the corresponding SD values are shown in a white box. "*" and "**" indicate significant differences between the last developmental stages at the level of 0.05 and 0.01, respectively. BY1 represents the C. sinensis cv. ‘BaiYe 1’, HJY represents the C. sinensis cv. ‘HuangJinYa’, SCZ represents the C. sinensis cv. ‘SuChaZao’, S1-S3 represent the three flower developmental stages. [file 12870_2022_3853_MOESM3_ESM.docx]

**Supplementary Fig. S3** Gene expression analysis of the three *C. sinensis* varieties during flower development. For each FPKM interval, the average number of expressed genes and the corresponding SD values are shown in a white box. "*" and "**" indicate significant differences between the last developmental stages at the level of 0.05 and 0.01, respectively. BY1 represents the *C. sinensis cv.* ‘Baiye 1’, HJY represents the *C. sinensis cv.* ‘Huangjinya’, SCZ represents the *C. sinensis cv.* ‘Suchazao’, S1-S3 represent the three flower developmental stages.
