# Supplementary material for: Transcriptome analysis reveals the roles of phytohormone signaling in tea plant (Camellia sinensis L.) flower development
Source: BMC Plant Biol. 2022 Oct 4;22:471. doi: 10.1186/s12870-022-03853-w (PMC9531472; doi:10.1186/s12870-022-03853-w)
Supplement: Supplementary file 4 — Additional file 4: Table S1: The primers used for qRT-PCR verification. [file 12870_2022_3853_MOESM4_ESM.docx]

**Supplementary Table S1** The primers used for qRT-PCR verification

| Gene | Forward primer (5´-3´) | Reverse primer (5´-3´) |
| --- | --- | --- |
| *β-actin* | GCCATCTTTGATTGGAATGG | GGTGCCACAACCTTGATCTT |
| *PHYA1 (TEA005460)* | TTGACTGTGGCTGTGAAG | CTTGATGAGGTTCCGATGT |
| *CO3 (TEA026771)* | GCAACTGTACCACTGAAGA | CGAATCCTTGGCTGACTAT |
| *GAI1 (TEA009882)* | CCTGGCTCGGAGAATCTA | TTGGTTGGCTGTGAAGTG |
| *VIP2 (TEA013506)* | AGAATGAAGATGGCAGAAGA | ATATGTGACTCCGATGTGAA |
| *VIL1 (TEA031773)* | TGGTCATTCGGAGGCTAA | GGCTGTTGTAGTCTTAGGTT |
| *MAF1 (TEA033019)* | ACAGTTACAATCCAGACTCT | CCTCGTAAGATACTCCATCA |
| *CTR1 (TEA002020)* | TCTATACCGCCTCATCCAT | CATACCTTCACCGTCCAAT |
| *AOC (TEA001905)* | AACTCACCACCACTACCA | CGCTCGTTGATCTCATACA |
| *PYL4 (TEA016225)* | GGTGGTTGTGGAGTCGTA | CTGAGCGAGCGATTGAAG |
| *DET2 (TEA020527)* | TCACTATGCCACCTACGA | GGACAACTCACAACCTCAA |
