# Supplementary material for: Transcriptome analysis reveals the roles of phytohormone signaling in tea plant (Camellia sinensis L.) flower development
Source: BMC Plant Biol. 2022 Oct 4;22:471. doi: 10.1186/s12870-022-03853-w (PMC9531472; doi:10.1186/s12870-022-03853-w)
Supplement: Supplementary file 5 — Additional file 5: Table S2: ESI-MS/MS parameters for determination of eight phytohormones. [file 12870_2022_3853_MOESM5_ESM.docx]

**Supplementary Table S2** ESI-MS/MS parameters for determination of eight phytohormones

| Phytohormones | Scan Mode | Parent ion  (m/z) | Daughter ion  (m/z) | Declustering potential (V) | Collision  Energy (V) |
| --- | --- | --- | --- | --- | --- |
| Gibberellic acid 1, GA_1_ | ESI^-^ | 347.4 | 259.2^*^/273.1 | -25 | -28/-16 |
| Gibberellic acid 3, GA_3_ | ESI^-^ | 345.2 | 143.0/239.2^*^ | -80 | -30/-33 |
| Jasmonic acid, JA | ESI^-^ | 209.2 | 58.9^*^ | -54 | -16 |
| Salicylic acid, SA | ESI^-^ | 137 | 92.9^*^/65 | -50 | -20/-39 |
| Abscisic acid, ABA | ESI^-^ | 263.1 | 153.0^*^/204.2 | -60 | -14/-27 |
| Gibberellic acid 4, GA_4_ | ESI^-^ | 331.4 | 243.2^*^/213.1 | -131 | -24/-39 |
| Trans-Zeatin riboside, TZR | ESI^+^ | 352.3 | 220.2^*^/136/202.1 | 90 | 25/40/32 |
| Indole-3-acetic acid, IAA | ESI^+^ | 176.2 | 129.8^*^/102.9 | 65 | 12/42 |

^*^, quantification ions
