# Supplementary material for: Transcriptome analysis reveals the roles of phytohormone signaling in tea plant (Camellia sinensis L.) flower development
Source: BMC Plant Biol. 2022 Oct 4;22:471. doi: 10.1186/s12870-022-03853-w (PMC9531472; doi:10.1186/s12870-022-03853-w)
Supplement: Supplementary file 6 — Additional file 6: Table S3: The parameters of gradient elution of HPLC. [file 12870_2022_3853_MOESM6_ESM.docx]

**Supplementary Table S3** The parameters of gradient elution of HPLC

| Time (min) | Flow rate (mL/min) | A% |
| --- | --- | --- |
| 0-6 | 0.3 | Decline from 90 to 10 |
| 6-9 | 0.3 | 10 |
| 9-9.1 | 0.3 | Increment to 90 |
| 9.1-16 | 0.3 | 90 |
