# Supplementary material for: Transcriptome analysis reveals the roles of phytohormone signaling in tea plant (Camellia sinensis L.) flower development
Source: BMC Plant Biol. 2022 Oct 4;22:471. doi: 10.1186/s12870-022-03853-w (PMC9531472; doi:10.1186/s12870-022-03853-w)
Supplement: Supplementary file 7 — Additional file 7: Table S4: Summary of RNA-Seq data and mapping metrics. [file 12870_2022_3853_MOESM7_ESM.docx]

| Sample ID | Total Raw Reads (Mb) | Total Clean Reads (Mb) | Total Clean Bases (Gb) | Clean Reads Q20 (%) | Clean Reads Q30 (%) | Clean Reads Ratio (%) | Total mapped Rate (%) | Uniquely mapped Ratio (%) |
| --- | --- | --- | --- | --- | --- | --- | --- | --- |
| BY1S1-1 | 52.50 | 51.31 | 7.70 | 97.39 | 93.70 | 97.74 | 90.96 | 84.64 |
| BY1S1-2 | 40.99 | 40.10 | 6.02 | 97.36 | 93.62 | 97.83 | 90.77 | 85.05 |
| BY1S1-3 | 56.22 | 54.95 | 8.24 | 97.33 | 93.55 | 97.73 | 91.47 | 85.07 |
| BY1S2-1 | 46.05 | 45.01 | 6.75 | 97.23 | 93.40 | 97.74 | 91.20 | 84.72 |
| BY1S2-2 | 54.04 | 52.85 | 7.93 | 97.30 | 93.54 | 97.80 | 91.34 | 84.72 |
| BY1S2-3 | 48.92 | 47.88 | 7.18 | 97.34 | 93.62 | 97.88 | 91.57 | 84.74 |
| BY1S3-1 | 57.91 | 56.49 | 8.47 | 97.17 | 93.35 | 97.55 | 90.21 | 83.23 |
| BY1S3-2 | 54.20 | 52.94 | 7.94 | 97.31 | 93.55 | 97.67 | 90.97 | 84.28 |
| BY1S3-3 | 48.47 | 47.35 | 7.10 | 97.10 | 93.15 | 97.70 | 90.49 | 83.21 |
| HJYS1-1 | 59.39 | 58.65 | 8.80 | 97.74 | 94.34 | 98.75 | 91.40 | 85.33 |
| HJYS1-2 | 55.12 | 54.47 | 8.17 | 97.76 | 94.39 | 98.82 | 91.22 | 85.15 |
| HJYS1-3 | 42.02 | 41.55 | 6.23 | 97.78 | 94.43 | 98.89 | 91.26 | 85.68 |
| HJYS2-1 | 55.11 | 54.42 | 8.16 | 97.59 | 94.06 | 98.75 | 91.27 | 85.14 |
| HJYS2-2 | 56.05 | 55.37 | 8.31 | 97.50 | 93.86 | 98.78 | 91.32 | 85.43 |
| HJYS2-3 | 55.82 | 55.14 | 8.27 | 97.58 | 94.04 | 98.78 | 91.51 | 85.49 |
| HJYS3-1 | 56.42 | 55.65 | 8.35 | 97.56 | 94.01 | 98.63 | 91.44 | 85.12 |
| HJYS3-2 | 52.91 | 52.20 | 7.83 | 97.04 | 92.81 | 98.66 | 90.74 | 84.38 |
| HJYS3-3 | 49.29 | 48.64 | 7.30 | 97.44 | 93.79 | 98.68 | 90.50 | 84.64 |
| SCZS1-1 | 56.81 | 55.96 | 8.39 | 97.71 | 94.28 | 98.51 | 91.17 | 85.21 |
| SCZS1-2 | 56.35 | 55.62 | 8.34 | 97.75 | 94.40 | 98.69 | 91.11 | 85.11 |
| SCZS1-3 | 62.34 | 61.40 | 9.21 | 97.70 | 94.28 | 98.49 | 91.28 | 85.36 |
| SCZS2-1 | 60.29 | 59.53 | 8.93 | 97.65 | 94.22 | 98.75 | 91.00 | 85.10 |
| SCZS2-2 | 63.84 | 62.99 | 9.45 | 97.60 | 94.10 | 98.68 | 91.06 | 84.98 |
| SCZS2-3 | 63.02 | 62.16 | 9.32 | 97.49 | 93.87 | 98.65 | 90.96 | 85.20 |
| SCZS3-1 | 55.48 | 54.76 | 8.21 | 97.66 | 94.21 | 98.70 | 91.08 | 85.02 |
| SCZS3-2 | 57.47 | 56.58 | 8.49 | 97.57 | 94.06 | 98.45 | 90.91 | 84.13 |
| SCZS3-3 | 55.49 | 54.74 | 8.21 | 97.52 | 93.94 | 98.64 | 90.66 | 84.44 |
| Average | 54.54 | 53.66 | 8.05 | 97.49 | 93.87 | 98.37 | 91.07 | 84.84 |

**Supplementary Table S4** Summary of RNA-Seq data and mapping metrics
